# Supplementary material for: Splice-Junction-Based Mapping of Alternative Isoforms in the Human Proteome
Source: Cell Rep. Author manuscript; Available in PMC 2020 Jan 15. (PMC6961840; doi:10.1016/j.celrep.2019.11.026)
Supplement: 2 [file NIHMS1546469-supplement-2.zip › DF1/PXD009737/TestisGluC/TestisGluC_7_DZIP1_QPAAPAVPMNAPALHTLETKSSLPMVHGRE.pdf]

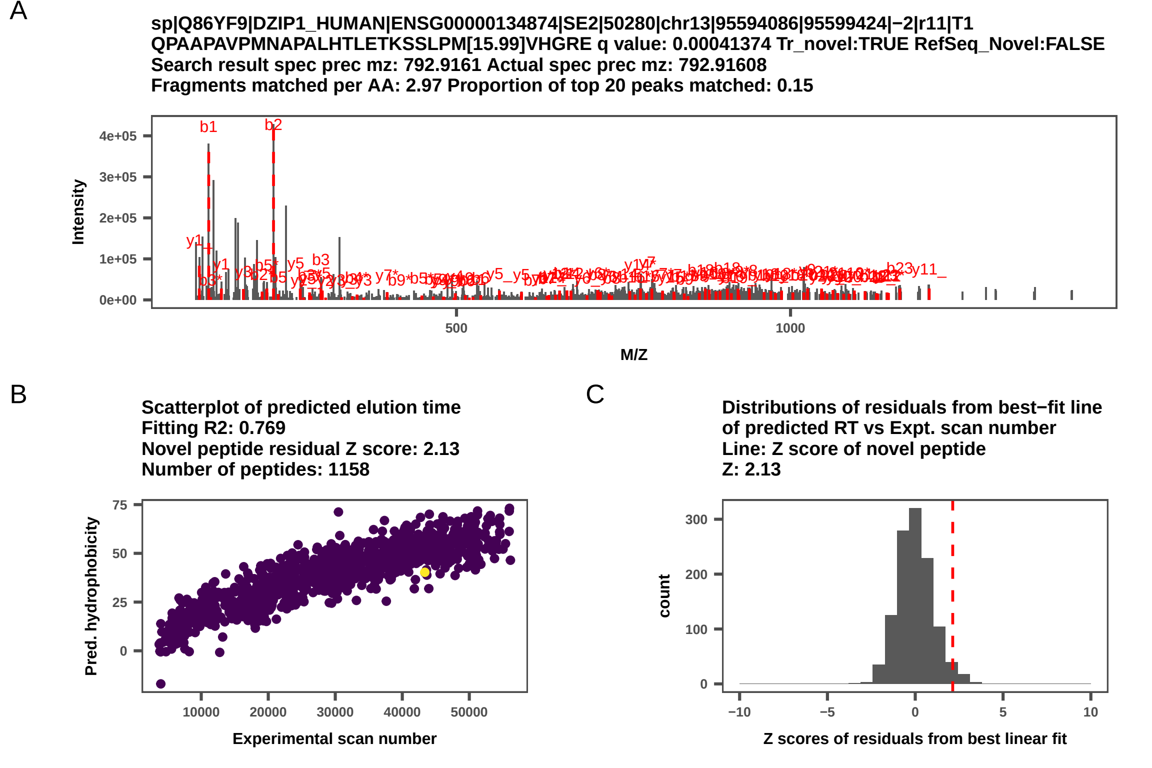
A
sp|Q86YF9|DZIP1_HUMAN|ENSG00000134874|SE2|50280|chr13|95594086|95599424|−2|r11|T1
QPAAPAVPMNAPALHTLETKSSLPM[15.99]VHGRE q value: 0.00041374 Tr_novel:TRUE RefSeq_Novel:FALSE
Search result spec prec mz: 792.9161 Actual spec prec mz: 792.91608
Fragments matched per AA: 2.97 Proportion of top 20 peaks matched: 0.15
Intensity
4e+05
3e+05
2e+05
1e+05
0e+00
b1
b2
y1
b3
y5
b23
y11_
500
1000
M/Z
B
Scatterplot of predicted elution time
Fitting R2: 0.769
Novel peptide residual Z score: 2.13
Number of peptides: 1158
Pred. hydrophobicity
75
50
25
0
10000
20000
30000
40000
50000
Experimental scan number
C
Distributions of residuals from best−fit line
of predicted RT vs Expt. scan number
Line: Z score of novel peptide
Z: 2.13
count
300
200
100
0
−10
−5
0
5
10
Z scores of residuals from best linear fit
